# The impact of digital healthcare on vulnerable pregnant women: A review of the use of the MyCare app in the maternity department at a central London tertiary unit

**DOI:** 10.3389/fdgth.2023.1155708

**Published:** 2023-04-21

**Authors:** Poppy Pierce, Melissa Whitten, Sara Hillman

**Affiliations:** ^1^ Medical School, Faculty of Medical Sciences, University College London, London, United Kingdom; ^2^ Women’s Health Division, Elizabeth Garrett Anderson Wing, University College London Hospital NHS Foundation Trust, London, United Kingdom; ^3^ Department of Maternal and Fetal Medicine, EGA Institute for Women’s Health, University College London, London, United Kingdom

**Keywords:** electronic health records systems, pregnancy, e-health, technology, vulnerable pregnant women, digital divide

## Abstract

**Introduction:**

Digitalisation offers innovative solutions within maternity services; however, vulnerable groups risk being overlooked. University College London Hospital's (UCLH) successful implementation of a digital maternity app, MyCare, gives women access to test results, information about appointments, and enables communication with healthcare professionals (HCPs). Yet, little is known about access and engagement among vulnerable pregnant women.

**Methodology:**

Research was conducted over a 3-month period (April–June 2022) in the Maternity Department at UCLH, UK. MyCare datasets were analysed, and anonymised surveys completed by vulnerable pregnant women and HCPs.

**Results:**

Lower rates of utilisation and engagement with MyCare were seen in vulnerable pregnant women especially among refugee/asylum seekers, those with mental health issues, and those facing domestic violence. Non-users were also more likely to be individuals from ethnic minority backgrounds, with a lower average social-deprivation-index decile, whose first language was not English, and with a significant history of non-attendance to appointments. Patient and HCP surveys highlighted various barriers to MyCare engagement, including a lack of motivation, limited language options, low e-literacy levels, and complex app interfaces.

**Conclusion:**

The use of a single digital tool, without a formulated pathway to identify and assist those not accessing or engaging with it, risks unequal care provision which may exacerbate health inequalities. This research advances the idea that digital exclusion is not necessarily a matter of *access to* technology, but an issue of a lack of *engagement with* these tools. Therefore, vulnerable women and HCPs must be integral to the implementation of digital strategies, to ensure no one is left behind.

## Introduction

To improve health outcomes and support national targets in the Long-Term Plan (1), the NHS has transitioned towards harnessing digital technology. The Maternity Transformation Programme (2) advocates for this as the main driving force in achieving the visions outlined in the Better Births Report (3), such as patient-centred care and tailored support for healthcare workers. The adoption of digital services has helped to streamline healthcare data, prioritise patient autonomy, encourage patient engagement, and empower both patients and healthcare providers (4). However, those who have most to gain from these provisions may often not be the people accessing them (5), and it is important to consider whether the digitalisation of healthcare may inadvertently perpetuate health inequalities for those at risk of poor health outcomes.

Research from Birth Companions revealed that pregnant women experiencing severe and multiple disadvantages are at a heightened risk for poor maternal and fetal health outcomes (6). Findings from the 2021 MBRRACE Maternal Report revealed that 8% of maternal deaths, documented in the UK between the years 2016–18, were of women facing ensuing social complexities, such as mental health diagnoses, substance misuse, and domestic violence (7). Vulnerability in pregnancy has been widely attributed to socioeconomic and ethnic inequalities (8), and as a result, the poorest maternal health outcomes are primarily seen among Black and South Asian women living in the most socially deprived areas (9). Social vulnerability is also correlated with higher rates of missed antenatal appointments (10, 11). Suboptimal antenatal care, alongside a myriad of other factors contributing to vulnerability, can lead to adverse outcomes such as the high rates of stillbirth seen in vulnerable women (12).

Pregnancy app usage has become routine in most women's maternity experience (13), emerging as primary tools for the provision of pregnancy information (14). Globally, pregnancy apps far outnumber other medical apps in the public domain (15), attesting to their appeal and popularity. While there is currently limited research to show the implications of pregnancy apps on maternal and fetal outcomes, in other medical contexts patient portal apps have been shown to “enhance the doctor-patient relationship, improve health status awareness, and increase adherence to healthcare in general” (16). It is still unclear, however, as to what extent patient portal apps address the needs of vulnerable women.

The most apparent barrier to the use of digital apps is the reliance on physical access to the internet and a smartphone. Furthermore, disparities in access and usage are compounded if individuals are then faced with language barriers or e-literacy difficulties. Existing research reveals that these barriers are notably more present among lower-socioeconomic and marginalised groups (17), alongside itinerant populations such as refugees, asylum seekers, and those experiencing homelessness (18). There is also robust evidence to show disabled people being significantly more at risk of digital exclusion (19). Additionally, there may be less engagement with apps from those who are generally less likely to engage with healthcare services, specifically those with mental health issues (20). Furthermore, studies show that those with lower education attainment levels, and those who are socially isolated and/or disabled, also express a lack of interest and negative attitudes towards the adoption of digital technology (19, 21).

This research sought to investigate the impact of digital health on vulnerable pregnant women in University College London Hospital (UCLH), a university-affiliated tertiary centre with a large and diverse patient population and approximately 6,000 births annually (22). In 2019, UCLH introduced an electronic health records system (EPIC), streamlining clinical health information. The maternity department became one of the first in the Trust to go entirely paperless when it piloted MyCare, the patient portal app tethered to EPIC. The intended rationale was to fulfil the needs of an already very engaged patient group, giving women access to test results, information about appointments and health-related updates, and enabling communication with healthcare professionals (HCPs). Attesting to its popularity, a recent audit revealed 97% of pregnant women had downloaded the app, but engagement levels or information about those who had not downloaded it was difficult to identify from routine reporting metrics.

## Methods and materials

This mixed-methods research was conducted over a 3-month period (April- June 2022) in the Maternity Department at UCLH, London, UK.

The overarching aims of this research were to quantify the use of MyCare, an electronic patient portal, among pregnant women, and to investigate whether any disparities existed relating to patients' socio-demographics or characteristics. This was evaluated through collection and analysis of datasets examining pregnant women's access to, and engagement with, MyCare.

In addition, this work aimed to gain insight into vulnerable pregnant women's views about using MyCare, and to identify the perceived utility of portal features alongside any potential barriers to adoption and engagement. The study also aimed to gain a broad overview of the HCP response to its implementation, noting any proposed improvements for the app. These insights were obtained through anonymised surveys completed by vulnerable pregnant women and HCPs.

To identify vulnerable pregnant women across all datasets, “vulnerability” was identified using the “Complex Social Factors in the Perinatal Period (Version 3)” UCLH guideline (23), adapted from the 2010 NICE guidelines on “Pregnancy and Social Factors” (24). The list of factors which would identify an individual as being vulnerable (vulnerability markers) includes women experiencing domestic violence; women with an existing enduring mental health illness; young women under the age of 19 years or with a history of having been in local authority care as a child; women who are currently involved with social services or have previously had children removed from their care; women who misuse substances (both alcohol and drugs); women with a learning disability; women with a physical disability; women who are homeless; women whose status is unclear, such as failed asylum seekers, recent migrants or women who have no recourse to public funds; women who have been trafficked or face abuse such as Modern Slavery; women who have difficulty reading or speaking English; and women involved with the criminal justice system.

### Dataset 1—engagement with MyCare

This dataset included all pregnant women booked into UCLH for their initial antenatal appointment in February 2022, identified through the EPIC platform. Age, parity, body mass index (BMI), ethnicity, and postcode data were collected from the patient's profile. Ethnicity was classified based on the ethnic groups identified in the 2021 Government Census (25). Postcodes were used to calculate the corresponding social deprivation index (SDI) deciles (26). These range from the most deprived 10% (Decile 1), to the least deprived 10% (Decile 10). Additional variables collected from the patient's profile were vulnerability (Y/N), with reasons for identified vulnerability (vulnerability markers); whether English was a first language, with “the need for an interpreter” used as a proxy for this; and whether MyCare had been downloaded, and corresponding engagement levels. Information regarding MyCare was established from the MyCare icon on the patient's profile. MyCare engagement was calculated by accessing an individual's MyCare usage data on their EPIC profile, and counting the number of messages read on the app. This was divided by the number of messages sent to the patient and recorded as a percentage. 50% was used as the arbitrary margin for categorising the level of engagement, high engagement being ≥50%, and low engagement being <50%. This metric was chosen as a measure of patient engagement, as messages sent to patients on MyCare are the main mechanism for receiving direct messages sent by HCPs but also for notifying patients about other information available to view on the app. This includes upcoming appointments, test results ready to be viewed, general information, health advice, and upcoming changes to service delivery. In addition to this metric, the number of messages sent by patients to HCPs was also determined.

### Dataset 2—MyCare not downloaded

This dataset included the medical record numbers of all women who received maternity care at UCLH after the implementation of MyCare in 2019, but who had never downloaded it during their pregnancy. The same variables were collected as in dataset 1, in addition to the number of non-attendances to appointments (DNAs), if any.

Individuals were excluded from further analysis if they had had an early miscarriage or termination of pregnancy, were transferred to another hospital, or if the patient had only attended one tertiary fetal medicine appointment and received their main antenatal care elsewhere.

### Data analysis of datasets 1 and 2

General trends were identified from datasets 1 and 2, through basic statistical interpretation and manipulation of raw data, using the Sort and Filter function on Microsoft Excel.

### Dataset 3—patient surveys

The patient survey primarily aimed to identify interest levels regarding the utility of MyCare among vulnerable pregnant women, and any associated challenges they may face whilst accessing or engaging with it.

The survey included 14 questions: dichotomous “yes” or “no” questions, 5-point Likert scale questions, checkbox questions, and open-ended questions with space for free text. The items (i.e., questions) in the survey asked general questions about English competency, access to technology, regular use of technology, and any previous usage of pregnancy apps/patient portals. Furthermore, specific questions about MyCare were asked such as: how they learnt about it; whether they had downloaded it; whether they received any support to download it; whether they would have liked more training on how to use it; what their interest levels towards it are and why; what their frequency of usage is; what their reason for usage based on app features; and if they had any suggestions for improvements, which would indirectly introduce any perceived barriers to usage.

A sample size was not predetermined. The intention was to cease recruitment of participants when data saturation was achieved. This was defined as no new sub-themes emerging from thematic analysis of the two open questions pertaining to interest levels and ways to improve the app. After completion of 22 surveys, it transpired that significant repetition of concepts was occurring; hence it was proposed that further sampling would not yield new insights addressing the primary aim of the patient surveys in identifying interest levels and any barriers.

Survey completion took place at UCLH on the maternity wards and within antenatal clinics. Purposive sampling was undertaken by selecting potential participants based on acquired knowledge of their characteristics and background. HCPs were asked to identify willing participants based on the following inclusion criteria:

i. Pregnant women currently booked into the UCLH Maternity Service and
ii. Older than 18 years and
iii. Vulnerable (defined using the “Complex Social Factors in the Perinatal Period (Version 3)” UCLH guideline (23).

### Dataset 4—HCP surveys

This survey aimed to assess HCPs' views towards the implementation of EPIC and MyCare, and to explore their involvement with MyCare development, noting any suggestions for optimising the utility of the app. It included 10 questions, with a mixed-style approach similar to that of the patient surveys above. The questions in the survey gathered information on their job title; the usability and usefulness of EPIC; whether they had seen MyCare from a patient's perspective; whether they ask for patient feedback, and how they process this information; how they think it can be improved to help vulnerable pregnant women; how they think it can be improved to help them deliver care; and how interested they are in the ongoing development of EPIC and MyCare within maternity care.

Opportunity sampling was employed by approaching all HCPs working within the Maternity Department who were willing and able to take part. Recruitment of participants was terminated when no new sub-themes emerged from qualitative analysis of the two open questions. These questions included the suggestions for improvement of MyCare for both vulnerable pregnant women and HCPs delivering care to this population.

For both HCPs and vulnerable pregnant women, paper-based surveys were distributed. Information about the research was verbally explained to participants, and this information was also available to read on the survey. For patients who required an interpreter, face-to-face translators were arranged. Participants were given time to read both the information about the research and the specific survey questions. They were then re-approached at a later point to obtain informed verbal consent and subsequently complete the survey.

### Data analysis of patient and HCP surveys

The variety of question types allowed for a mixture of qualitative and quantitative data to be obtained. Ordinal and nominal data were analysed using frequency statistics, through the Sort and Filter function on Microsoft Excel. The responses to the open questions were analysed using an inductive thematic approach to establish commonly communicated answers. Researchers read responses and made notes independently before defining preliminary codes. These codes were then developed and applied to the open-question text. This content analysis encompassed Braun and Clarke's 6-step framework (27).

### Ethical considerations

This project did not require UCLH ethical approval as it was classified as a service evaluation. This being said, procedures for fully informing participants, obtaining informed verbal consent, and ensuring anonymity, complied with UCLH ethical standards. Participants were made aware that their participation was entirely voluntary.

## Results

### Dataset 1—engagement with MyCare

Dataset 1 shows a sample of the pregnant women booked in for maternity care at UCLH during the month of February 2022. Prior to data analysis, 14 individuals were excluded (8 miscarried early, 3 had a termination of pregnancy, and 3 had transferred care to another hospital). The remaining population group (*N* = 636) were divided into 3 categories dependent on their MyCare status and activity: (1) ≥50% engagement, (2) <50% engagement, and (3) MyCare not downloaded. For those with MyCare downloaded, the average number of messages sent to patients was 40, with the average number of messages sent from patients to HCPs being 0.48. Engagement with MyCare was assessed through the percentage of messages read by the patient out of the total number of messages received. Participants were notified on their phone, or other specified device, if they received a message on MyCare.

Table 1 shows the sociodemographic characteristics and vulnerability markers for Categories 1, 2, and 3.

**Table 1:** The sociodemographic characteristics and vulnerability markers for categories 1, 2, and 3.

	Total (N)	People with or without MyCare downloaded (%)	MyCare users differentiated by engagement levels (%)	People in each category with vulnerability markers (%)
**Dataset 1**	**636**	**–**	**–**	**–**
MyCare Downloaded	597	93.9	**–**	**–**
≥50% Engagement (Category 1)	524	**–**	87.8	**–**
* Average Age (years)*	33	**–**	**–**	**–**
* Average Parity (n)*	0.94	**–**	**–**	**–**
* Average BMI (kg/m^2^)*	24.8	**–**	**–**	**–**
* Vulnerable*	103	**–**	**–**	19.7
*English not first language*	128	**–**	**–**	24.4
* Average SDI decile*	4.75	**–**	**–**	**–**
<50% Engagement (Category 2)	73	**–**	12.2	–
* Average Age (years)*	34	–	–	–
* Average Parity (n)*	1.47	–	–	–
* Average BMI (kg/m^2^)*	25.8	–	–	–
* Vulnerable*	19	–	–	26.0
* English not first language*	17	–	–	23.3
* Average SDI decile*	4.76	–	–	–
MyCare Not Downloaded (Category 3)	39	6.1	–	–
* Average Age (years)*	30	–	–	–
* Average Parity (n)*	1.78	–	–	–
* Average BMI (kg/m^2^)*	25.5	–	–	–
* Vulnerable*	19	–	–	48.7
* English not first language*	19	–	–	48.7
* Average SDI decile*	3.72	–	–	–

Across Categories 1 and 2 the average age was similar (33 and 34 respectively), whereas Category 3 had a lower average age (30).

There was a link between a higher average parity and lower engagement or non-use of MyCare, with the average parity being 0.94, 1.47, and 1.78 in categories 1, 2, and 3 respectively.

There was no clear relationship between MyCare engagement and average BMI across the 3 categories, with BMI being 24.8, 25.8, and 25.5 in categories 1, 2, and 3 respectively.

There was an association between higher rates of vulnerability and lower engagement or non-use of MyCare, with the percentage of vulnerable individuals being 19.7%, 26%, and 48.7% in categories 1, 2, and 3 respectively.

Mental health issues, alongside physical and learning disabilities, were the most prevalent vulnerabilities within the low engagement category (Category 2). Mental health issues, domestic violence, and refugee/asylum seeker status were the most prevalent vulnerabilities in the group who did not have MyCare (Category 3).

The percentages of women whose first language was not English were similar across the high and low engagement categories (24.4% and 23.3% respectively), however, the percentage was considerably higher for those without MyCare (48.7%).

The average SDI deciles were similar across the high and low engagement categories (4.75 and 4.76 respectively), however, for those without MyCare, the average SDI decile was lower (3.72).

In terms of ethnicity, white women made up the greatest proportion of the high engagement category (58.2% of Category 1), whereas all ethnic groups combined (Asian, Black, Mixed, or other ethnic background) made up most of the low engagement category (57.5% of Category 2). In those who did not have MyCare (Category 3), the majority were White Jewish (46.2%), warranting a distinction from those of a white background (7.6%). The remainder were predominantly those from an ethnic background (Asian, Black, or other ethnic background) (46.2%).

### Dataset 2—MyCare not downloaded

In dataset 2 there were 499 women in the cohort, of which 249 were excluded for the following reasons: 169 attended only one fetal medicine appointment, 10 were identified as having since downloaded MyCare, 33 miscarried early, 27 had transferred care to another hospital, and 10 had a termination of pregnancy.

Of the 250 women who were included in the dataset, 51.2% were White Jewish, with the remainder being predominantly from an ethnic background (Asian, Black, Mixed, or other ethnic background) (40%). The average age was 30.6, the average parity was 2.49, and the average BMI was 26.7.

Out of the 250 women, 151 (60.4%) had a history of DNAs, from anywhere between 2 and 22 DNAs to antenatal appointments.

For the proportion of those who were White Jewish (*n* = 128), HCPs explicitly outlined in patient notes that they did not have MyCare due non-ownership of a smartphone for religious reasons. Further analysis excluded this cohort and only included those who did not have a clearly documented reason for non-use of MyCare (*N* = 122).

Further analysis of sociodemographic characteristics and vulnerability markers was carried out on this remaining cohort (*N* = 122), as seen in Table 2.

**Table 2:** The sociodemographic characteristics and vulnerability markers for those without a documented reason for MyCare non-use.

	Total (N)	Percentage of total (%)
** **	**122**	–
Average Age (years)	30.9	–
Average Parity (n)	2.32	–
Average BMI (kg/m^2^)	26.9	–
Vulnerable	98	80.3
English not first language	81	66.4
DNA (High non-attendance rate)	88	72.1
Ethnic Minority	99	81.1
Average SDI decile	3.85	–

The average age was 30.9, the average parity was 2.32, and the average BMI was 26.9.

Vulnerability, English not being a first language, and being from an ethnic minority background were all factors associated with non-use of MyCare. Refugee/asylum seeker status, mental health issues, and domestic violence were identified as the most common vulnerabilities. For the 88 women with a history of DNAs, 38% had patient notes outlining that they were unaware of their appointments. Of this, 63% had corresponding notes on EPIC from HCPs stating, “appointments sent via MyCare”, despite the EPIC MyCare icon showing that the patients did not have MyCare.

### Patient surveys

A total of 22 patient surveys were completed with vulnerable pregnant women, with 11 requiring an interpreter. All participants, except one, had access to a phone or tablet, with 17 stating that they are regular users of different apps (non-pregnancy related) on their phone. 20 had never used a pregnancy app or patient portal before.

All women had MyCare downloaded except one who did not have a phone. Participants were made aware of MyCare through a midwife/nurse (*n* = 17), doctor (*n* = 3), or friend/family member (*n* = 2). Independent of face-to-face antenatal appointments, 16 participants received all other information regarding their maternity care (e.g., upcoming appointment reminders, test results, advice etc.) solely through MyCare. Alongside this, 3 others stated that they also received this information through text messages. 3 participants answered “other”, giving the following responses: “I rely on my memory but I have missed a few appointments”, “sometimes they will print out my next appointment and any other information for me”, and “they usually call me to let me know about my appointments and tests”.

Only 4 participants stated that they had received support from a midwife or doctor in setting up and using MyCare, with 10 stating that they would have benefited from receiving more thorough training.

12 of women showed interest in using MyCare, with 2 having neutral feelings, and 8 not being interested. An open question was asked to ascertain why, with Table 3 showing the themes identified from their answers.

**Table 3:** Table showing the themes, sub-themes, and example quotations, that represent participants’ reasoning behind their interest levels towards using MyCare.

Themes	Sub-themes	Examples of Participant Responses
Positive	Convenience and Usefulness	*“I can keep track of my appointments”*
	Communicate with HCP	*“Can speak to HCP for advice when I’m at home”*
Negative	Lack of motivation	*“Even if I had a phone I would not use it, it doesn’t benefit me in any way and its difficult to use”* *“I have no intention to use it, I downloaded it during my first appointment and haven’t logged in since. I don’t think I even remember my password”* *“I have logged in a few times, but I’m not really bothered to use it”*
	Limited Language Options	*“It does not have my language”* *“Requires husband support when using the app because he speaks English”*
	Confusing Interface and Information	*“Confusing app features”* *“Clinic codes are confusing because it does not explain where my appointment is or what it is for”*
	Complex App Journey	*“Difficult to set it up yourself”* *“Login process is complex”* *“Need pin number if locked out—hard to get back in”*
	Privacy Concern	*“I do not trust it with my personal information”*
	Poor Communication Channel	*“Not all information is streamlined, get some info on email and some on app so it becomes confusing”*
Neutral	Preference for Non-Digital	*“Even if it had my language, I probably still would not use it, I prefer not to use apps”* *“Wish there was paper option, letter, don’t really care about app and they are frustrating”*

#### Positive themes

The most common positive theme pertained to the usefulness of MyCare as a tool to view appointments and keep track of maternity care. Only one said that it enables them to communicate with HCPs to ask for advice.

#### Negative themes

Several negative themes emerged, such as a lack of motivation to use the app; limited language options available, where in a few cases the woman's husband, being the only English speaker in the family, operated it on their behalf; the difficult app interface, for example, the use of clinic codes that are not user-friendly; and the complexity of the system, for example, login codes and pin numbers were brought up as intricacies of the app that were confusing and difficult to navigate. In addition, privacy concerns were expressed, stemming from distrust in inputting personal details into the app.

#### Neutral themes

Several participants expressed a preference for non-digital resources, citing that even if certain barriers were overcome, they would still not use MyCare.

Most women used MyCare only when the app sent them a prompt or when they were reminded by a midwife/doctor (*n* = 16). 3 said they used it weekly and 3 stated that they had never used it.

Those who had never used it (*n* = 3) were excluded from further participation in the survey: one did not have MyCare due to not having a phone, one did not use it due to privacy concerns, and one said she has no intention to ever use it. The survey was terminated here for these participants as subsequent questions asked about their experience using MyCare, of which they could not contribute insights as they had never used it.

Of the remaining 19 women surveyed, all had used MyCare to view appointments, with 11 having used it to view test results, and a very small proportion having used it to access health information (*n* = 2) and communicate with HCPs (*n* = 1). Almost all women said the app could be improved (*n* = 18). Thematic analysis of the open text revealed that 9 participants agreed that MyCare could be easier to use, alongside 10 stating that it did not have enough language options.

### HCP surveys

A total of 62 surveys were carried out with HCPs working within the Maternity Department. Midwives made up 58.1% of the cohort, with doctors 27.4%, and nurses, student midwives, pharmacists, and administration staff making up the remainder.

91.9% of HCPs said that EPIC is easy to use, with all respondents finding EPIC and MyCare to be helpful in the delivery of their care. 30 HCPs answered the follow-up question as to why, with 46.7% stating that it allowed them to have greater engagement with their patients, and 53.3% stating that it enabled better distribution of information to patients.

Despite this, 87.1% of HCPs had never seen MyCare from the patient perspective. Furthermore, 6 HCPs said they asked patients for feedback, with 5 of these stating that they did not know who to report this information back to. Of those who did not ask patients for feedback regarding MyCare, 7 said this was not necessary given that they worked on a labour ward, 3 said this was not a requirement of their role (1 pharmacist, 1 hospital administrator, and 1 nurse), and 2 said it was redundant, given that there is an in-app function for obtaining feedback from patients. The remaining 44 did not give a reason as to why they did not ask patients for feedback about MyCare.

HCPs were asked to what extent they thought MyCare was suitable as a platform for vulnerable pregnant women to use. 10 HCPs expressed that MyCare was not suitable at all (16.1%), alongside 32 stating it was suitable some of the time (51.6%). In contrast, 11 expressed that MyCare was suitable most of the time (17.7%), yet 0 stated that it was suitable all the time. 9 were impartial (14.5%).

Two open questions were merged and thematically analysed. One related to suggestions for improvement of MyCare for vulnerable pregnant women, and the other related to suggestions to help make MyCare more suitable for HCPs to deliver care to this population. Table 4 shows the themes identified from the text.

**Table 4:** Table showing the themes, subthemes, and selected quotations that represent participants’ suggestions for MyCare improvement.

Themes	Subthemes	Quotation
Improvements internal to MyCare	Welfare concern alert function	*“Incorporate a way for them to report domestic violence or concerns discreetly”* *“Create a clear point of contact, with better access to a nominated person, clear indication of emergency numbers provided”* *“Include an alert button so if they feel they need to speak to someone but do not know how then they can press the button and it calls through to an emergency helpline”*
	Multiple language options	*“Include different languages”*
	Varied formats for displaying information	*“Put more information in pictorial form”*
	Simpler login and password reset	*“Patients get locked out, make it easier to get back in”* *“No need for username access”*
	Simpler interface	*“Appointment and location clearer for patients (specify phone numbers, floor level, point of contact) do not use clinic codes as they are not user-friendly”* *“Less options/buttons as so much on there it is sometimes hard to find things for patients”*
	Clear patient identifiable numbers	*“Have the patient's MRN easily accessible when opening the app. Helps us to pull out their notes when they call in or arrive at hospital”* *“Make it easier to find NHS number”*
Improvements external to MyCare	App training for patients	*“Training for women regarding how to use the app”*
	Available alternatives	*“Make app/version of it usable on non-smartphones”* *“Victims of DV may have their phone watched/controlled by their partner, have alternative modes instead of app”* *“If patient declines to use the app/ or cannot access it then they should be given the option for paper records”*
	Provide digital tools to facilitate app use	*“No phone, no internet, provide resources”*
	App training for HCPs	*“I do not have much interaction with patient-facing side of app and would like to see what it looks like from this perspective”* *“There is in fact a facility within MyCare to download the app in certain languages. But this is dependent on the device you are using. Many have no idea that it is possible, healthcare professionals and patients alike”*

#### Improvements internal to the app

Common suggestions for improvements internal to the app included more language options and configuring the interface and applying features that enable easier navigation. For example, by making the login process simpler and replacing clinic codes for appointments with more useful user-friendly information. Other suggestions were to display hospital numbers on the main page of MyCare and incorporate a method for patients to promptly alert staff of welfare concerns.

#### Improvements external to the app

For improvements external to the app, HCPs suggested creating alternatives that operate on non-smartphones, and alternatives that are entirely non-digital. In addition, further training for both HCPs and patients was emphasised to encourage better implementation and engagement with MyCare.

Lastly, 64.5% of HCPs said that they would be interested in being involved in the ongoing development of EPIC and MyCare for maternity care.

## Discussion

MyCare has rapidly become the primary channel through which women can access antenatal services in a busy tertiary London maternity hospital. However, data shows some poor engagement and lack of access in vulnerable groups. It can be inferred from our findings that MyCare gives an additional advantage to those who are already able to engage with maternity care. Exclusive digitisation risks overlooking vulnerable women, who are more likely to be in greater need. Steps must be taken to ensure that digital interventions are inclusive of all women and truly fit for purpose.

### Principal findings from datasets 1 and 2

The datasets illustrated a strong association between vulnerable pregnant women and lower rates of utilisation and engagement with MyCare, with the most prominent vulnerabilities emerging as those with refugee/asylum seeker status, those with mental health issues, and those facing domestic violence. Poor engagement with MyCare was also linked to those with disabilities, corroborating findings from studies that show this group to be at risk for digital exclusion (19). In addition, non-users of MyCare were more likely to be from ethnic minority backgrounds, with a lower average SDI decile, whose first language was not English, and with a significant history of non-attendance to antenatal appointments. These factors have all been robustly linked to adverse health outcomes and inequalities (8, 28, 29).

Across both datasets, White Jewish women made up a large proportion of individuals not using or engaging with MyCare. Studies show that the Orthodox Jewish community has less exposure to technology as their culture can impose restrictions on the possession of smartphones (30). This was a recognised barrier and documented by HCPs, but also demonstrates the need to pay closer attention to the needs of different communities when creating digital tools.

Interestingly, there was no documentation of the reason for any other population that did not have MyCare. As a result, HCPs were sending information and appointment details through the app to those who did not even have it downloaded. Correspondingly, patients reported not knowing they had appointments booked. Notably, a strong link between MyCare non-use and DNAs emerged in the data, consistent with studies showing higher rates of missed appointments among non-patient portal users (31). In addition, studies show non-attendance to antenatal appointments as an indicator of vulnerability (10, 11) and have been associated with adverse pregnancy outcomes (32).

In addition, whilst vulnerability and digital exclusion were shown to be intrinsically linked, they were not mutually exclusive. Individuals who are not engaging with digital tools, irrespective of whether they are vulnerable or not, may experience disruptions in care, as seen in the high non-attendance rates shown in the results. As a result, they may be at greater risk of poorer health outcomes (33). This will become more pertinent if digital tools begin to replace, instead of complement, current services, particularly if there are not sufficient alternatives in place for those who are not accessing them. Such criticisms validate arguments for digital technology heightening existing social inequalities for those who are already vulnerable (34), but it may also create further disparities of its own.

### Patient and HCP surveys

Levels of interest in MyCare were polarised among vulnerable pregnant women. In practice, higher levels of interest did not translate to high engagement levels.

When considering the reasons why people did not engage with MyCare, physical barriers, such as ownership of a smartphone, appeared to have an even smaller impact than speculated in previous research (35). The primary barriers to use pertained to patients' lack of willingness and motivation. These barriers may be mitigated by evaluating the usability of the app (36).

Findings have corroborated existing research that shows complex app interfaces and limited language options to be significant barriers to use of digital tools (17, 37). Findings revealed that some husbands were using and monitoring MyCare on the woman's behalf because they were the only ones who could understand English. By not satisfying language requirements, confidentiality is compromised, which has been linked to negative health outcomes (38). Although the manufacturer of MyCare states that other languages are available, this is not a recognised function in the current set-up and HCPs feel unqualified to offer it, as evidenced in survey responses.

Some negative perceptions towards MyCare due to privacy concerns were revealed. Notably, fears regarding data sharing have been shown to be more prevalent among ethnic minorities (39) and have been shown to influence engagement with healthcare (40). HCP communication skills may be pivotal in addressing these concerns, by facilitating patient use of MyCare alongside enhancing patient trust in providers.

In addition, participants expressed criticism of the app's complex interface. Promoting engagement relies on providing training to women. However, a lack of engagement may also relate to the usability of the MyCare platform, in that the diverse needs of vulnerable women have not been considered in app design and delivery of information about how to use it (41). Despite MyCare having an extensive range of functionality, in practice vulnerable groups are only accessing a small percentage of this.

HCPs play an important role in the implementation of MyCare, however, the findings suggest that they may not realise how significant their influence can be on patient engagement. Most HCPs had never seen the app from the patient perspective, nor had they ever asked patients about their experiences and concerns regarding it. This is a very strong indicator that the promotion for engagement should start with HCPs. HCP advocacy can influence patient behaviour by increasing their “knowledge, confidence, and self-determination” (42). This can provide patients with the tools to feel empowered to take an active role in their own care. HCPs need to spend more time promoting the benefits of MyCare to patients, anticipating issues that might arise, and emphasising confidentiality of data and autonomy. Clear communication with patients, alongside listening to their concerns, has the potential to reduce disparities in patient usage of these digital tools (43).

Giving women the physical tools to access digital tools is ineffective as a standalone solution if barriers such as a lack of motivation; limited knowledge of, or confidence in, digital tools; limited language options; low e-literacy skills; complex app interface, and usability are not considered. This research advances the idea that digital exclusion is not necessarily a matter of *access to* technology, but an issue of a lack of *engagement with* these tools (44).

### Limitations

The views expressed in the patient surveys may not be generalisable, as the small sample is unlikely to reflect the full spectrum of vulnerable patients. However, these findings do not necessarily need to be statistically generalisable for researchers to draw inferences that can advance and improve MyCare in ways that support vulnerable pregnant women. Furthermore, as the research was based at UCLH, the findings may not be applicable to other health care settings. Additionally, the use of SDI as an indicator for deprivation was not an absolute measure. It does not capture whether a specific individual is deprived. For example, in datasets 1 and 2, several pregnant refugees had an SDI of 7+, as they had been placed in temporary hotels in less deprived areas. SDI was therefore used as a general measure of social deprivation and results should be interpreted with this knowledge in mind. Lastly, it is not possible to establish a causal relationship between the observed associations as there is a potential for unmeasured confounding variables. Factors such as the duration of UK residence, health literacy, and education attainment were not collected, all of which may influence MyCare engagement.

### Suggestions for further research

To build upon the research, additional parameters of MyCare engagement could be measured through internal metrics derived from the app itself to decipher whether similar trends emerge for individuals engaging with the app in other ways. Furthermore, the tangible impact of MyCare and how it relates to targeted health behaviours and patient-centred outcomes should be evaluated. In addition, further reviews should be conducted on patient portals used in other hospitals and any emerging trends and findings should be compared.

### Implications and clinical relevance

One important finding was that increased knowledge and advocacy of MyCare among HCPs would potentially encourage greater uptake and engagement in vulnerable populations. Therefore, it might be relevant to facilitate training that encompasses learning about MyCare from the patients' perspective, alongside incorporating information on how to solicit feedback from patients and who to report this to. The findings also demonstrate an interest among vulnerable pregnant women for the implementation of digital tools within maternity care. An adapted version of MyCare, considering all the pragmatic and culturally sensitive suggestions highlighted in the surveys, has the potential to foster uptake and engagement. In addition, further strategies to engage vulnerable women could include training sessions early in antenatal care that clearly clarify the role of MyCare in maternity care, how best to utilise it, and to identify and address any potential issues that may dissuade patients from engaging. Overall, the success of MyCare and digital health requires synergy between HCPs and patients as empowering both groups is critical in realising any benefits associated with patient portals.

## Conclusion

Digital tools, such as the MyCare patient portal, offer opportunities to reduce exclusion through wider provision of information, greater interactive contact with HCPs, and the opportunity for self-management of care. Yet the use of a single digital tool, without a formulated pathway to identify and assist those not accessing or engaging with it, risks unequal care provision which may exacerbate health inequalities. The results of this research demonstrate that giving women the physical tools to download the app is ineffective as a standalone solution if other barriers limit their ability to use and engage with it. Therefore, vulnerable women and HCPs must be integral to the implementation of digital strategies, to ensure no one is left behind.

## Data Availability

The raw data supporting the conclusions of this article will be made available by the authors, without undue reservation.

## References

[1] NHS England. The NHS Long Term Plan. London (2019). Available at https://www.longtermplan.nhs.uk/wp-content/uploads/2019/08/nhs-long-term-plan-version-1.2.pdf (Accessed June 9, 2022).

[2] NHS England. Maternity Transformation Programme. England.nhs.uk. (2022). Available at https://www.england.nhs.uk/mat-transformation/ (Accessed June 9, 2022).

[3] NHS England. Better Births. (2016). Available at: https://www.england.nhs.uk/wp-content/uploads/2016/02/national-maternity-review-report.pdf (Accessed June 9, 2022).

[4] Imison C Castle-Clarke S Watson R Edwards N. Delivering the benefits of digital health care. Nuffield Trust. (2016). Available at: https://www.nuffieldtrust.org.uk/files/2017-01/delivering-the-benefits-of-digital-technology-web-final.pdf

[5] Tudor Hart J. The inverse care law. Lancet. (1971) 297(7696):405–12. 10.1016/S0140-6736(71)92410-X 4100731

[6] Thomson G Balaam M Lessen L Austin J Jenkins H Burnell J Birth Companions Research Project: Experiences and Birth Outcomes of Vulnerable Women. (2016). Project Report. Birth Companions, London, UK. Available from: https://www.researchgate.net/publication/326607034_Birth_Companions_Research_Project_Experiences_and_Birth_Outcomes_of_Vulnerable_Women_Project_Report_Birth_Companions_London_UK” https://hubble-live-assets.s3.amazonaws.com/birth-companions/file_asset/file/96/Birth_Companions_Research_Project_UCLan.pdf

[7] Knight M Bunch K Tuffnell D Patel R Shakespeare J Kotnis R (eds.) Saving lives, improving mothers’ care—lessons learned to inform maternity care from the UK and Ireland confidential enquiries into maternal deaths and morbidity 2017–19. Oxford: National Perinatal Epidemiology Unit, University of Oxford (2021). Available at: https://www.npeu.ox.ac.uk/assets/downloads/mbrrace-uk/reports/maternal-report-2021/MBRRACE-UK_Maternal_Report_2021_-_FINAL_-_WEB_VERSION.pdf

[8] Fernandez Turienzo C Newburn M Agyepong A Buabeng R Dignam A Abe C Addressing inequities in maternal health among women living in communities of social disadvantage and ethnic diversity. BMC Public Health. (2021) 21(1):176. 10.1186/s12889-021-10182-4 33478445 PMC7817762

[9] Jardine J Walker K Gurol-Urganci I Webster K Muller P Hawdon J Adverse pregnancy outcomes attributable to socioeconomic and ethnic inequalities in England: a national cohort study. Lancet. (2021) 398(10314):1905–12. 10.1016/S0140-6736(21)01595-6 34735797

[10] Lewis G (ed). The Confidential Enquiry into Maternal and Child Health (CEMACH). Saving Mothers' Lives: reviewing maternal deaths to make motherhood safer - 2003-2005. The Seventh Report on Confidential Enquiries into Maternal Deaths in the United Kingdom. (2007). London: CEMACH. Confidential Enquiry into Maternal and Child Health. Available from: https://www.publichealth.hscni.net/sites/default/files/Saving%20Mothers%27%20Lives%202003-05%20.pdf

[11] Brygger Venø L Pedersen L Søndergaard J Ertmann R Jarbøl D. Assessing and addressing vulnerability in pregnancy: general practitioners perceived barriers and facilitators—a qualitative interview study. BMC Primary Care. (2022) 23(1):142. 10.1186/s12875-022-01708-9 PMC9164392 35659201

[12] Heazell A Budd J Smith LK Li M Cronin R Bradford B Associations between social and behavioural factors and the risk of late stillbirth—findings from the Midland and North of England Stillbirth case-control study. BJOG. (2021) 128(4):704–13. 10.1111/1471-0528.16543 32992405

[13] Frid G Bogaert K Chen K. Mobile health apps for pregnant women: systematic search, evaluation, and analysis of features. J Med Internet Res. (2021) 23(10):e25667. 10.2196/25667 34524100 PMC8561408

[14] Brown H Bucher T Collins C Rollo M. A review of pregnancy apps freely available in the google play store. Health Promot J Austr. (2019) 31(3):340–2. 10.1002/hpja.270 31225924

[15] Tripp N Hainey K Liu A Poulton A Peek M Kim J An emerging model of maternity care: smartphone, midwife, doctor? Women Birth. (2014) 27(1):64–7. 10.1016/j.wombi.2013.11.001 24295598

[16] Carini E Villani L Pezzullo AM Gentili A Barbara A Ricciardi W Boccia S. The impact of digital patient portals on health outcomes, system efficiency, and patient attitudes: updated systematic literature review. J Med Internet Res. (2021) 23(9):e26189. 10.2196/26189 PMC8459217 34494966

[17] Hughson JP Daly JO Woodward-Kron R Hajek J Story D. The rise of pregnancy apps and the implications for culturally and linguistically diverse women: narrative review. JMIR Mhealth Uhealth. (2018) 6(11):e189. 10.2196/mhealth.9119 30446483 PMC6269626

[18] Huxley CJ Atherton H Watkins JA Griffiths F. Digital communication between clinician and patient and the impact on marginalised groups: a realist review in general practice. Br J Gen Pract. (2015) 65(641):e813–21. 10.3399/bjgp15X687853 26622034 PMC4655735

[19] Pérez-Escolar M Canet F. Research on vulnerable people and digital inclusion: toward a consolidated taxonomical framework. Univ Access Inf Soc. (2022). 10.1007/s10209-022-00867-x PMC8808275 35125988

[20] Anderson CM Robins CS Greeno CG Cahalane H Carr Copeland V Marc Andrews R. Why lower income mothers do not engage with the formal mental health care system: perceived barriers to care. Qual Health Res. (2006) 16(7):926–43. 10.1177/1049732306289224 16894224

[21] Helsper E Reisdorf B. The emergence of a “digital underclass” in Great Britain and Sweden: changing reasons for digital exclusion. Sage J. (2016) 19(8):1253–70. 10.1177/1461444816634676

[22] Why choose UCLH: University College London Hospitals NHS Foundation Trust. (2022). Available at: https://www.uclh.nhs.uk/our-services/find-service/womens-health-1/maternity-services/why-choose-uclh (Accessed June 9, 2022).

[23] Driver T Owino J Smith P. Standard operating procedure. Safeguarding in the maternity unit guidelines. London, United Kingdom: University College London Hospital (2020). (Accessed June 9, 2022).

[24] National Institute for Health and Care Excellence (NICE) clinical guideline [CG110]. Pregnancy and complex social factors: A model for service provision for pregnant women with complex social factors. NICE. (2010). Available at: https://www.nice.org.uk/guidance/cg110 (Accessed June 9, 2022). 31855334

[25] List of ethnic groups. GOV.UK. Available at: https://www.ethnicity-facts-figures.service.gov.uk/style-guide/ethnic-groups (Accessed January 30, 2023).

[26] The English Indices of Deprivation 2019 (IoD2019). Assets.publishing.service.gov.uk. (2019). Available at: https://assets.publishing.service.gov.uk/government/uploads/system/uploads/attachment_data/file/835115/IoD2019_Statistical_Release.pdf (Accessed June 9, 2022).

[27] Braun V Clarke V. Using thematic analysis in psychology. Qual Res Psychol. (2006) 3:77–101. 10.1191/1478088706qp063oa

[28] Thomson K Moffat M Arisa O Jesurasa A Richmond C Odeniyi A Socioeconomic inequalities and adverse pregnancy outcomes in the UK and republic of Ireland: a systematic review and meta-analysis. BMJ Open. (2021) 11:e042753. 10.1136/bmjopen-2020-042753 33722867 PMC7959237

[29] Sentell T Chang A Ahn HJ Miyamura J. Maternal language and adverse birth outcomes in a statewide analysis. Women Health. (2016) 56(3):257–80. 10.1080/03630242.2015.1088114 26361937 PMC4868388

[30] Barzilai-Nahon K Barzilai G. Cultured technology: the internet and religious fundamentalism. Inf Soc. (2005) 21(1):25–40. 10.1080/01972240590895892

[31] Graham TAD Ali S Avdagovska M Ballermann M. Effects of a web-based patient portal on patient satisfaction and missed appointment rates: survey study. J Med Internet Res. (2020) 22(5):e17955. 10.2196/17955 PMC7267992 32427109

[32] Raatikainen K Heiskanen N Heinonen S. Under-attending free antenatal care is associated with adverse pregnancy outcomes. BMC Public Health. (2007) 7:268. 10.1186/1471-2458-7-268 17900359 PMC2048953

[33] Why digital inclusion matters to health and social care. NHS Digital. NHS. Available at: https://digital.nhs.uk/about-nhs-digital/corporate-information-and-documents/digital-inclusion/digital-inclusion-in-health-and-social-care#:∼:text=In%20an%20increasingly%20digital%20world,the%20skills%20to%20use%20it (Accessed March 21, 2023).

[34] Van Deursen AJAM Van Dijk JAGM. Internet skill levels increase, but gaps widen: a longitudinal cross-sectional analysis (2010–2013) among the Dutch population. Inf Commun Soc. (2015) 18(7):782–97. 10.1080/1369118x.2014.994544

[35] Honeyman M Maguire D Evans H Davies A. Digital technology and health inequalities: a scoping review. (2020) Cardiff: Public Health Wales NHS Trust. Available at: https://phw.nhs.wales/publications/publications1/digital-technology-and-health-inequalities-a-scoping-review/ (Accessed January 30, 2023).

[36] Health inequalities and mitigating risks of Digital Exclusion. Good Things Foundation. (2022). Available at: https://www.goodthingsfoundation.org/insights/health-inequalities-and-mitigating-risks-of-digital-exclusion/ (Accessed January 30, 2023).

[37] Baldwin JL Singh H Sittig D Davis Giardina T. Patient portals and health apps: Pitfalls, promises, and what one might learn from the other. Healthc (Amst). (2016) 5(3):81–85. 10.1016/j.hjdsi.2016.08.004 27720139 PMC8386919

[38] Gerrish K Chau R Sobowale A Birks E. Bridging the language barrier: the use of interpreters in Primary Care Nursing. Health Soc Care Community. (2004) 12(5):407–13. 10.1111/j.1365-2524.2004.00510.x 15373819

[39] Tsatsou P. Vulnerable people’s digital inclusion: intersectionality patterns and associated lessons. Inf Commun Soc. (2021) 25(10):1475–94. 10.1080/1369118X.2021.1873402

[40] Stablein T Lorenzo Hall J Pervis C Anthony D. Negotiating stigma in health care: disclosure and the role of electronic health records. Health Sociology Review. (2015) 24(3):227–41. 10.1080/14461242.2015.1078218

[41] Goedhart NS Zuiderent-Jerak T Woudstra J Broerse JEW Betten AW Dedding C. Persistent inequitable design and implementation of patient portals for users at the Margins. J Am Med Inf Assoc. (2021) 28(2):276–83. 10.1093/jamia/ocaa273 PMC7883982 33463691

[42] Chen J Mullins CD Novak P Thomas SB. Personalized strategies to activate and empower patients in health care and reduce health disparities. Health Educ Behav. (2016) 43(1):25–34. 10.1177/1090198115579415 PMC4681678 25845376

[43] Lyles C Schillinger D Sarkar U. Connecting the dots: health information technology expansion and health disparities. PLoS Med. (2015) 12(7):e1001852. 10.1371/journal.pmed.1001852 26172977 PMC4501812

[44] Anthony DL Campos-Castillo C Lim PS. Who isn't using patient portals and why? evidence and implications from a national sample of US adults. Health Aff (Millwood). (2018) 37(12):1948–54. 10.1377/hlthaff.2018.05117 30633673

